# Interaction and integration among behaviors of adult *Drosophila* in nature

**DOI:** 10.1371/journal.pone.0278427

**Published:** 2023-07-13

**Authors:** Jeannette Silva-López, Pablo Godoy, Lilian Jara, Raúl Godoy-Herrera

**Affiliations:** 1 Facultad de Ciencias, Departamento de Ciencias Básicas, Universidad del Bío-Bío, Chillán, Chile; 2 Faculty of Veterinary Medicine, University of Montreal, Saint-Hyacinthe, Canada; 3 Facultad de Medicina, Programa de Genética Humana, Universidad de Chile, Santiago, Chile; Biomedical Sciences Research Center Alexander Fleming, GREECE

## Abstract

Living in environments whose ecologies vary in periods as short as 24 h is a challenge for animals as *Drosophila* species that inhabit pear and apple orchards. These orchards have sunny and shady sections. The size and shape of these habitats change daily according to the position of the sun in the sky. Sunny areas are related to dryness and water loss, and shady places have lower temperatures and higher humidity. The presence of heterospecific flies may lead to competition for space and food. In sunny habitats we did not find adult *Drosophila*. In shady sections we found conspecific groups *D*. *melanogaster*, *D*. *simulans*, *D*. *immigrans*, *D*. *subobscura*, and the Chilean endemic *D*. *pavani* perched on grasses and herbs at 8–10 cm from fruits that had fallen on the ground. In the fruits, 99% of the adults were females and they were not grouped. The way in which daily changes in the size and shape of shady habitats together with the presence of heterospecific adults influence the selection of places to live is poorly understood in *Drosophila*. Our experiments show that adults of the five species prefer dark areas. The experimental results show that the odors of each species: i) influence conspecifics to select similar perch sites and decrease mobility, and ii) increase mobility in heterospecific adults and modify their perch site preferences. Attractions between conspecifics, the repulsions between species, and preferences for shaded areas matter in choosing a place to live in the five *Drosophila* species. These behaviors seem to have evolved as coordinated routines, contributing to the coexistence of the five *Drosophila* species in the apple and pear orchards examined.

## Introduction

Animal species regulate their activities in response to environmental changes, with consequences for individual biological fitness [1–3]. For example, changes in temperature, humidity and acidity/alkalinity on grape grains (*Vitis vinifera*) and prickly pear fruits (*Opuntia ficus-indica*), modulate egg-laying site selection in *Drosophila melanogaster* and *Drosophila simulans* [4]. In some species of vertebrates, environmental changes may impact their behavior, including social relationships. Thus, chemical changes caused by anxiolytic benzodiazepine dumped into rivers obstructed social recognition, feeding and mating in the fish *Perca fluviatilis* [5]. In this same context, some species of birds emit vocal signals to overcome communication problems originated by urbanization, so that they can be heard by conspecifics in noisy environments [6].

Therefore, natural environments provide circumstances for animals to exhibit behaviors that often differ from those shown in the laboratory. For example, peak activity in adult *D*. *melanogaster* occurs at dawn and dusk in the laboratory, while they are diurnal rather than crepuscular in the wild [7]. Similarly, golden hamsters are nocturnal in the laboratory and diurnal under natural conditions [8]. Sea lamprey pheromones are required for mating in the laboratory, but in the wild such substances do not induce searching for partners [9]. The feeding behavior displayed by the hummingbird *Selaphorus rufus*, takes on all importance in nature. Adult *S*. *rufus* relates flower appearance to the volume and concentration of nectar, as these birds scan this source of food [10]. Therefore, it is essential investigate animal behavior in nature to understand well the ecology and evolution of animal species [11–14].

Consistent with the changing and variable environments where they live, larval and adult *Drosophila* possess sophisticated sensory receptors and brain structures suggesting that they can make a diversity of timely decisions in the wild [15]. However, studies on interactions and links among routines involved in food acquisition, eggs-laying site selection and/or allocation of space in the wild are not well-described in the *Drosophila* literature, principally because most ecological, population genetics, and neurobiological studies have been conducted in the laboratory [16,17], and are rarely documented in nature. The Markow [18], Dukas [19] and Reaume and Sokolowski [20] studies are exceptions.

Living in fruit orchards is a challenge for *Drosophila* species. Such orchards comprise sunny and shady habitats. Sunny areas tend to be dry, and shady places have lower temperatures and higher humidity. Daily, changes in the sun’s position modify the size and shape of sunny and shady habitats, and heterospecific adults may compete for shady areas. We found that adults of *Drosophila melanogaster*, *Drosophila simulans* (Subgenus *Sophophora*, species group *melanogaster*), *Drosophila immigrans* (Subgenus *Drosophila*, species group *immigrans*), *Drosophila subobscura* (Subgenus *Sophophora*; species group *obscura*), and *Drosophila pavani* endemic to Chile. (Subgenus *Drosophila*, species group *mesophragmatica*) form conspecific groups in shady sites in fruit orchards. We studied in the nature and laboratory the contribution of adult nonsexual behavior of the five species to the occupation of shady habitats. We found that the adults preferred dark places to stay and that odors emitted by them attracted conspecifics and repelled heterospecific. To live in variable and changing shady habitats of fruit orchards, adults of all five *Drosophila* species integrate preferences for dark areas and responses to odors emitted by them.

## Materials and methods

### Stocks and fruit orchards

We collected adults and established strains from natural populations of cosmopolitan *D*. *melanogaster*, *D*. *simulans*, *D*. *subobscura*, *D*. *immigrans*. We also tested natural populations of *D*. *pavani*, a South American neotropical species, endemic to Chile. Together with an additional 9 to 12 species, *D*. *pavani* forms the *mesophragmatica* group of species of *Drosophila* [21–23]. We conducted ethological observations and collections of the five species between March–April (2018) late summer early autumn, in two Chilean localities: i) Chillán 36° 36’ 00”S and ii) Quillón 36° 44’ 00”S. There is approximately 44 km between Chillán and Quillón. We selected two fruit orchards in each locality: i) a pear (*Pyrus communis*, winter Nelis variety) orchard and ii) an apple (*Malus domestica*, Fugi variety) orchard. The distance between the fruit orchards was approximately 2 km in each locality. In the pear and apple orchards, we found that fruits fallen on the ground were in different stages of fermentation. In concordance with abundance of the decaying fruits Chilean *Drosophila* populations reach maximum size in the autumn [22]. We formed a strain/species/orchard/locality. That is, four strains per species, achieving a study of 20 strains in total. We examined such a number of strains and species because we wanted to know if daily changes in lighting conditions in fruit orchards affected all *Drosophila* species equally. The five species have among themselves different degrees of relatedness [23].

Each strain was founded with 12 males and 12 females. Stocks were all reared under lights on continuously at 18.0 ± 1.0°C and 80.00% humidity in 250 cc glass bottles on synthetic Burdick’s medium [24]. Facilities to change the light/dark cycle were not available in the laboratory. The life cycle duration of *D*. *pavani* and *D*. *immigrans* is approximately twice as that of *D*. *melanogaster*, *D*. *simulans* and *D*. *subobscura* at 18.0°C [21]. We conveniently coordinated the culture tasks to collect adults of the five species within a maximum period of a week. The adults tested in the experiments corresponded approximately to the fourth generation of breeding in the laboratory. All adults of the strains and species were vigorous, actively moving, and female fecundities were similar as suggested by the abundant offspring.

### Rainfall and temperature in the orchards

In Chillán, annual the annual rainfall is approximately 872 mm, and in Quillón it is approximately 1000 mm; the annual mean temperatures are 13.3°C (Chillán) and 14.9°C (Quillón). The climate in Chillán and Quillón is Mediterranean (Dirección Metereológica de Chile: https://climatologia.meteochile.gob.cl/ applicatio/ publicaciones/ reporteEvolucionClima).

### Illumination conditions in the orchards

**Fig 1:**
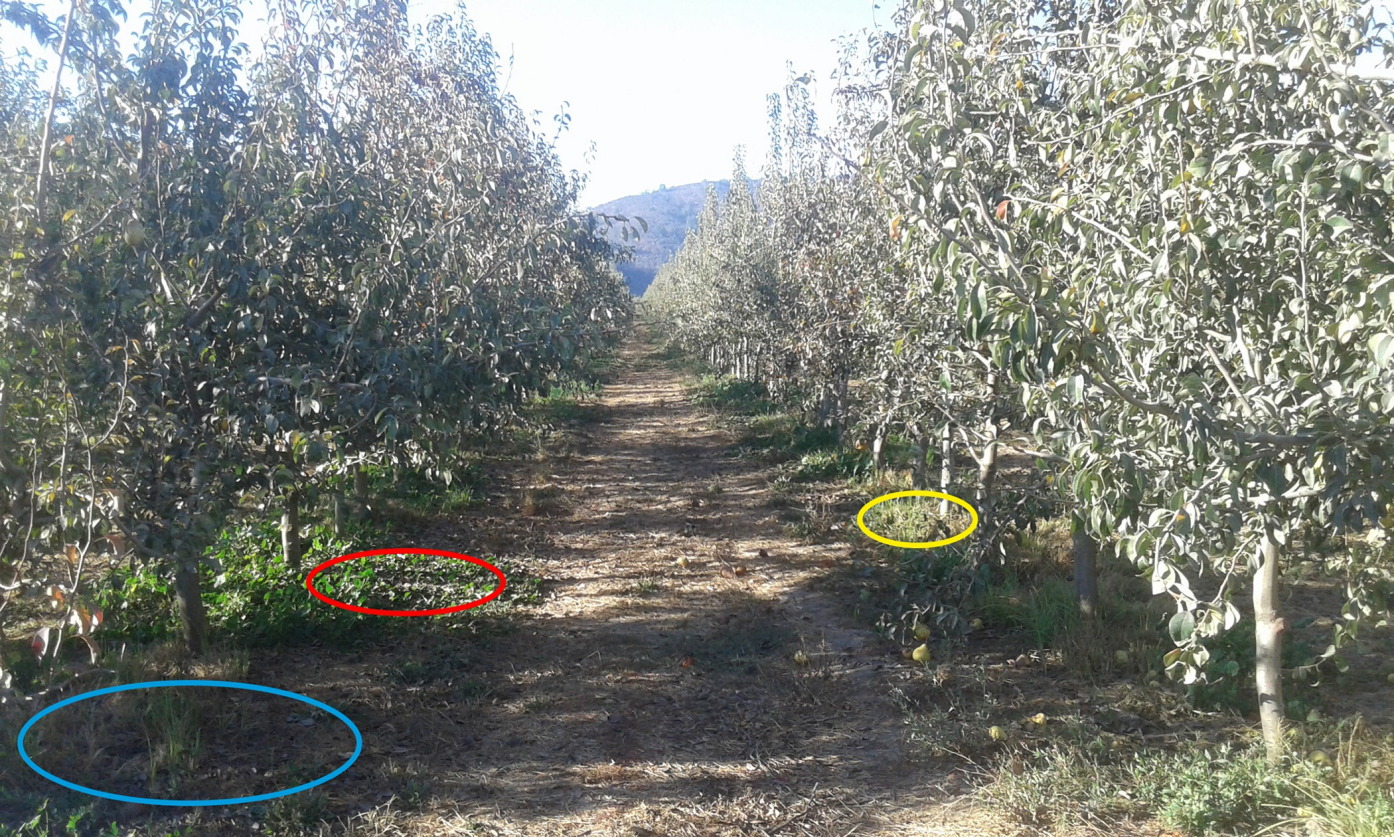
A pear orchard in Quillón. Blue ellipse, shady habitat; red ellipse, sunny/shady habitat; yellow ellipse, sunny habitat. Similar habitats were detected in the apple orchards in Chillán and Quillón. Photograph taken at 6.20 pm (see Materials and Methods).

In the orchards there were microhabitats that differed in illumination, and they were classified as sunny and shady habitats (Fig 1). We also detected that the sun’s rays were projected onto the ground through the canopy of the trees creating areas that were less illuminated than the sunny places, but more illuminated than the shady sections; those sites were called sunny/shady habitats (Fig 1). The size and shape of the three habitats daily changed according to sun position in the sky. We measured the amount of light in the three habitats between 12:00 and 14:30 (n = 40 measurements/microhabitat/orchard/locality). We used a Leitz-Westlar Microsix-L 2875 photometer and a random number table to select the sites to perform the records [25]. During that same period of time, we recorded temperatures in sunny and shady habitats. We used a Braun PTR 2000 maximum and minimum thermometer.

### Fruit collections

We recorded the number of fruits fallen on the ground under the canopy of n = 40 trees/orchard /locality (Chillán and Quillón). We carefully inspected the individual fruits searching for adult *Drosophila*. Subsequently, we collected at random n = 5 pears (apples) from each of n = 40 fruit trees/orchard/locality. Each collected fruit was weighed using a Sartorius CP 225D balance.

### Adult *Drosophila* in the fruit orchards

**Fig 2:**
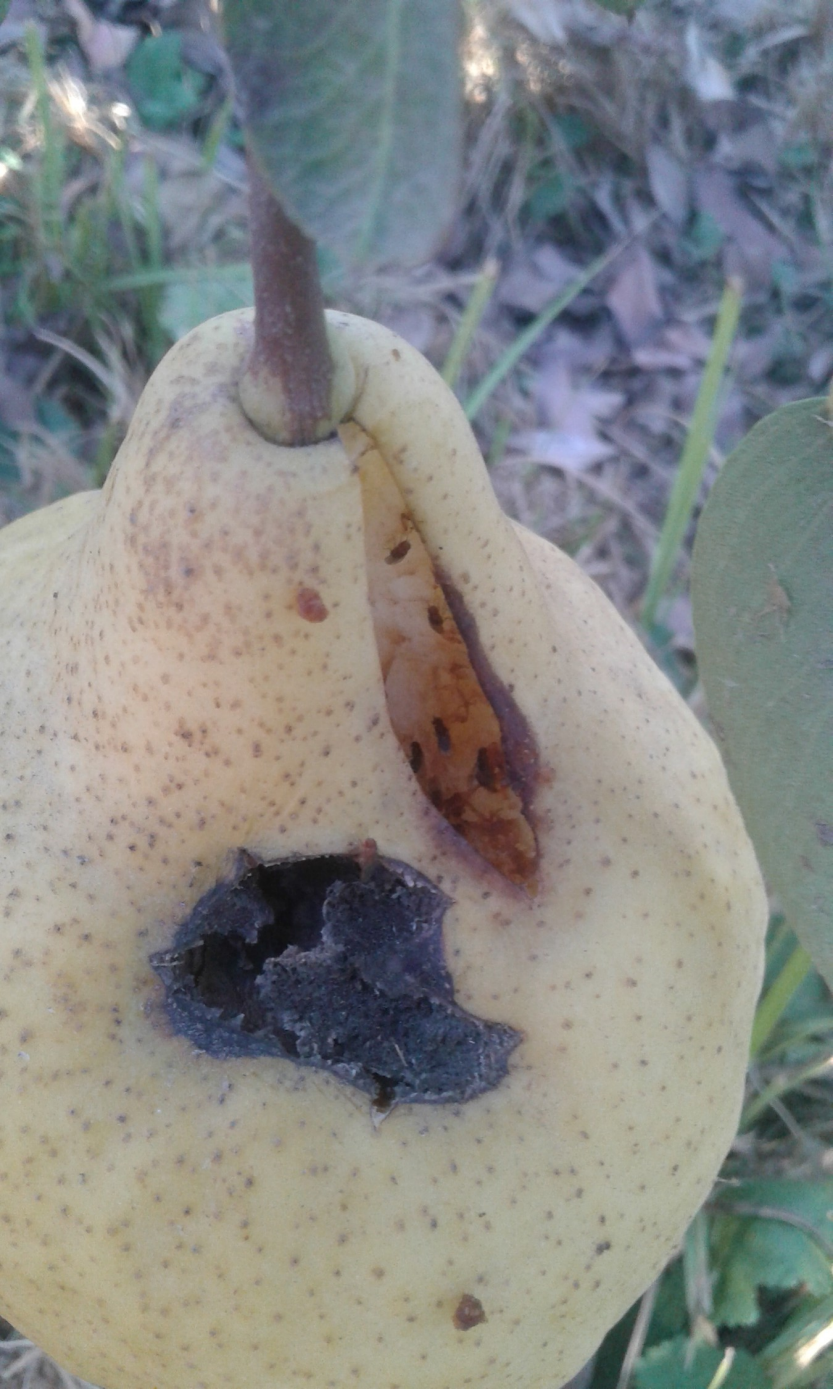
A pear fruit fallen on the ground. The fruit shows a fissure. In the fissure there are 6 adults. Taxonomic classification indicated that they corresponded to *D*. *simulans*. Another adult *Drosophila* is emerging from a black orifice in the fruit.

**Fig 3:**
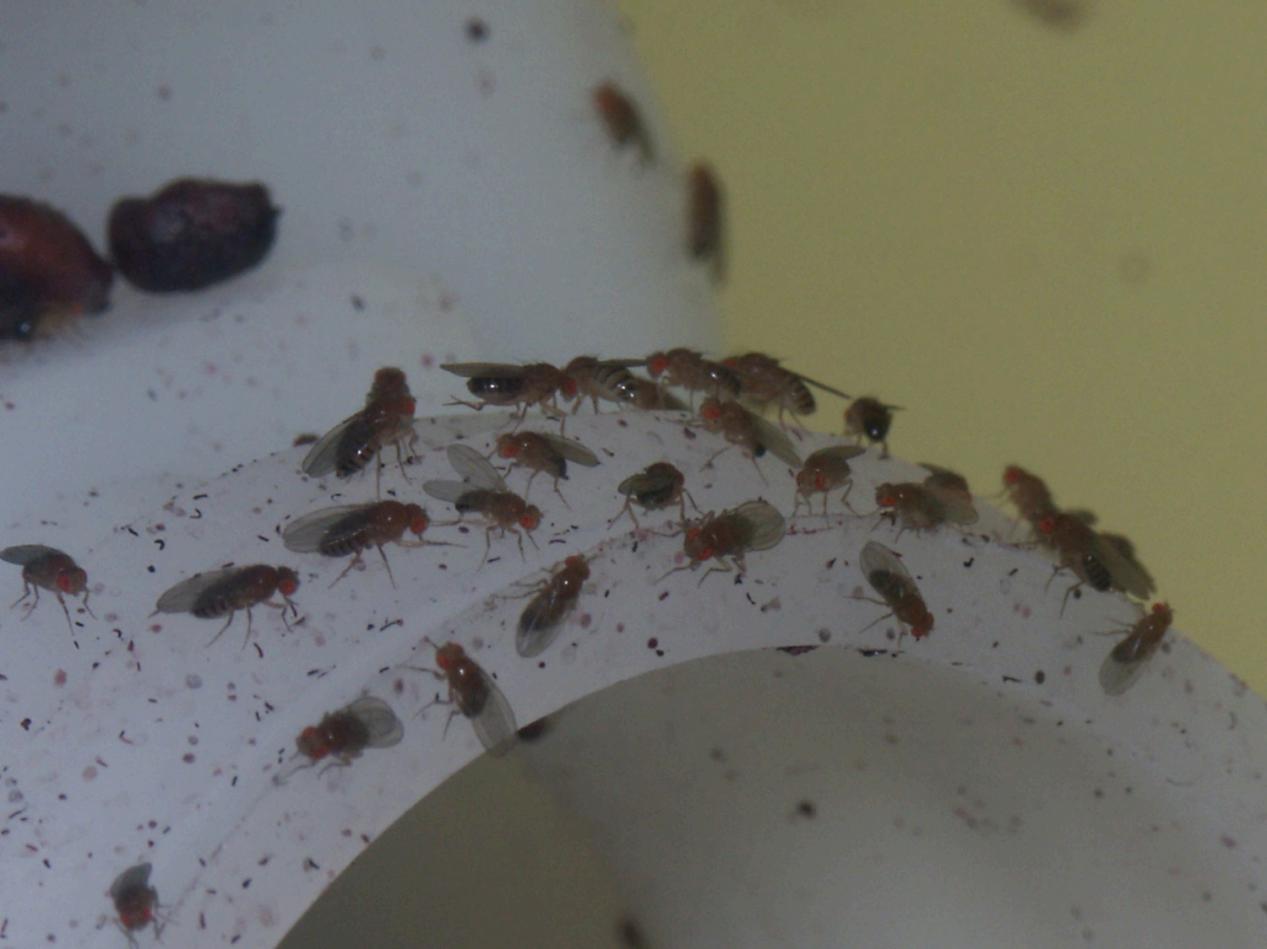
A conspecific cluster of adult *Drosophila* in a wine cellar. Males and females can be observed. The flies were on a machine used to make wine (see Materials and Methods). Taxonomic classification indicated that the flies belonged to *D*. *melanogaster*. On grasses and herbs, we observed similar conspecific groups (S2 Table).

We used our portable telephones (iPhone XR iOS14.1) at maximum magnification to take photographs of fruits fallen on the ground. Approximately 80.00% of the fruits had fissures. In these crevices we found adult *Drosophila* (Fig 2). Most of the imagoes were grouped on grasses and herbs near the fruits that had fallen to the ground (see Results). Due to the poor contrast of the flies against grasses and herbs, we could not obtain reliable images with our portable telephones. There were wine cellars one block away from the apple and pear orchards. In the buildings we detected groups of adult *Drosophila* similar to those observed in the fruit orchards, and illumination conditions were apt to take photographs (Fig 3). Identifying the species in shady habitats was extremely difficult. We used a glass aspirator to carefully collect the fly clusters (*D*. *melanogaster*, *D*. *simulans*, *D*. *subobscura*, *D*. *immigrans* and *D*. *pavani*) observed on grasses and herbs. Each fly group was deposited into a 39.5 cm^**3**^ labeled vial filled with 2.5 cm^**3**^ of *Drosophila* Burdick’s medium [24]. We also carefully collected the adults found in the fissures of the fruits fallen on the ground. By using Brncic keys [26] and a Leica M7 stereoscopic microscope, we taxonomically classified the imagoes deposited in each vial. We did not determine whether the flies were virgins. In the fruit orchards, we also looked for aggressive encounters between imagoes, similar to those reported in the laboratory [27]. The observations in the fruit orchards were performed in 1 h-windows between 9:00 am and 6:00 pm.

### Experiments

The experiments were designed to better understand three phenomena related to the behavior of adult *Drosophila* in fruit orchards that came to our attention: i) permanency in habitats with poor illumination, ii) propensity to stay near conspecifics, and iii) tendency to remain immobile (Fig 3).

The experiments were performed at 20°C and 80% humidity between 9.30 and 12.30 am. For each treatment we recorded n = 50 individuals sex/strain/species/fruit orchard/locality. To reduce the experimental error, the records were made alternately at random. For this we use a table of random numbers [25]. For example, we recorded the behavior of a male (or female)/Quillón strain/*D*. *melanogaster*/pear orchard/Quillón locality. Then we recorded the behavior of a female (or male)/Chillán strain/*D*. *subobscura*/apple orchard/Chillán locality, until completing n = 50 specimens/strain/species/locality. Each author collected data in the absence of the other authors. Each team member recorded at least n = 50 specimens/sex/species/locality.

For the experiments we used 9.0 x 1.5 cm (diameter x height) Petri dishes. Each fly tested was aspirated individually into the respective Petri dish through a 0.5 cm diameter hole made in the wall of Petri dish. The hole was stoppered with a cotton wool plug to prevent flies escaping from the dish. Each Petri dish was used for one individual. We made the records 10 min after we introduced a specimen in a Petri dish. This period was sufficient for the flies to acclimate to the boxes [4]. Before performing the experiments, we checked the homogeneity of illumination conditions by using a Leitz-Westlar Microsix-L 2875 photometer. In the case of *D*. *melanogaster*, *D*. *simulans* and *D*. *subobscura*, adults tested were 4–5 days old postemergence. For *D*. *immigrans* and *D*. *pavani*, adults were 12–14 days old. In these two *Drosophila* species sexual maturity is reached 12 days post adult emergence from pupae [21].

### Preferences for dark and illuminated sites

**Fig 4:**
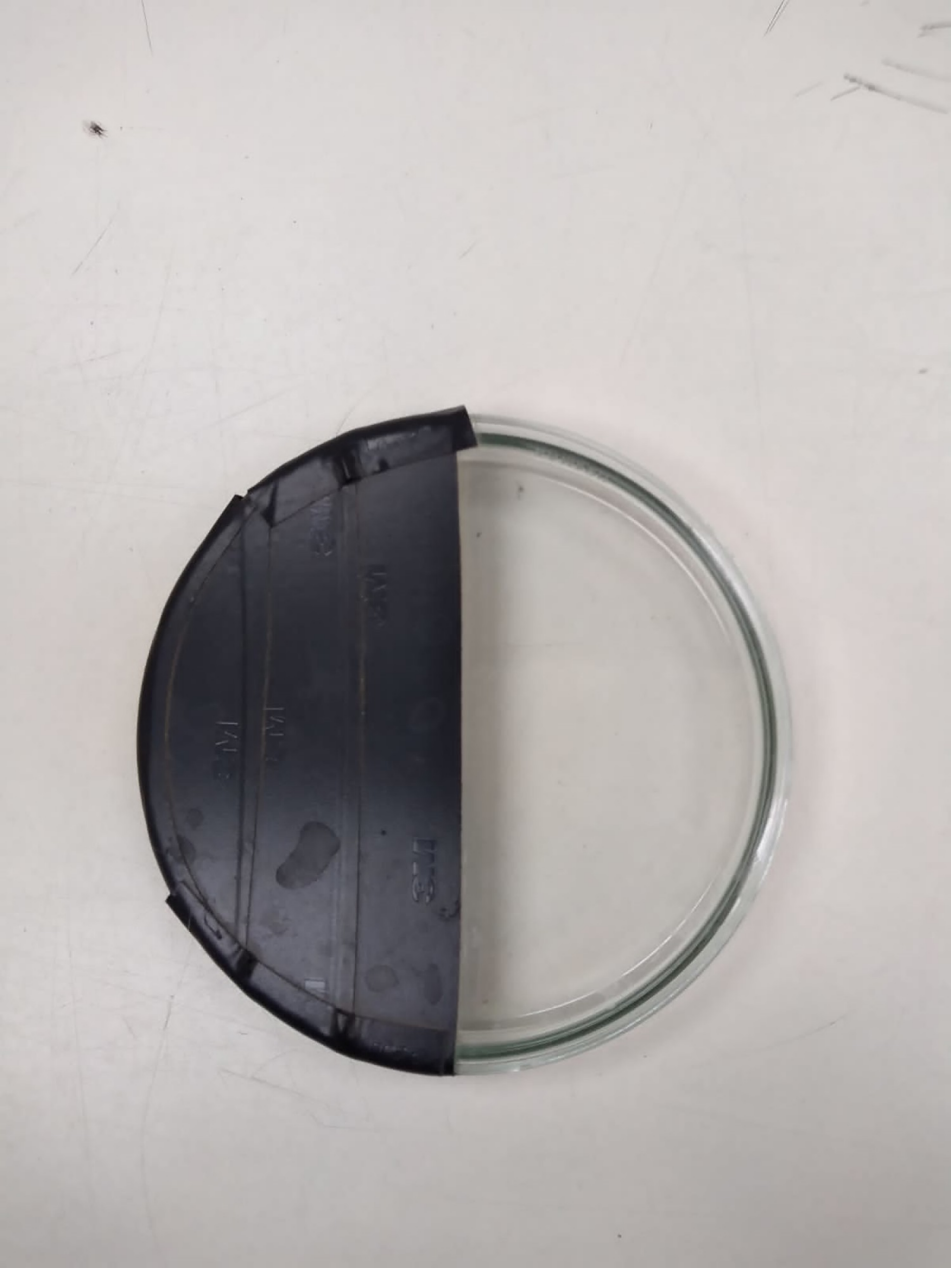
Experimental design to study adult response to illumination conditions in *Drosophila*. The boxes had three places that differed in illumination: i) a dark area, ii) an illuminated zone, and iii) an interface sector between the dark and illuminated areas. The flies were individually introduced into the box (see Materials and Methods).

In the fruit orchards, adults of the five *Drosophila* species were in shady habitats, suggesting preferences for dark sites. We tested this conjecture by wrapping half of a glass Petri dish in thick black paper as described above (Fig 4). The lids of these Petri dishes prevented the illuminated area of the boxes from being warmer than the dark zone, without an established temperature gradient at the dividing line [28,29]. Consequently, each Petri dish had an illuminated zone and a dark sector produced by the shadow the black paper cast on the Petri dish (Fig 4). The edge of the black paper deflected the rays of light, creating an interface zone between the dark and lighted areas of the box (Fig 4). Experiments were performed in a 2 x 3 m dark room to avoid interference from other light sources. The adults found in the dark zone presumably exhibited photonegative behavior. We assumed that the flies detected in the illuminated area had photopositive behavior. We also recorded the number of imagoes in the interface zone. We also compared the results obtained by darkening the right side of each box with those obtained by darkening the left side of the boxes. In both cases individuals tested were always found in the dark side of the boxes. We tested males and females of the strains formed with adults collected in the pear orchard (Chillán locality).

### Perch and moving site preferences in Petri dishes with conspecific and strange odors

In the fruit orchards the adults were perched nearby conspecifics away from other *Drosophila* species (Fig 3). Conspecific odors and odors of other *Drosophila* species might be involved in the behavior [4,12,30–32]. We used the Petri dishes described above to test whether conspecific odors and odors of another *Drosophila* species influenced perching site preferences and mobility. The capsules offered to the adults three sections to perch and move: ceiling, wall and floor.

In one treatment, 4 nonvirgin males and 4 nonvirgin females *D*. *melanogaster*, *D*. *simulans*, *D*. *immigrans*, *D*. *subobscura* and *D*. *pavani* were deposited, each species, into their respective Petri dish for 30 min. Four nonvirgin adults of each sex composed the clusters observed in grape orchards [4]. After 30 min, we removed the flies from the Petri dish, and one male (or female) of the same strain and species was aspirated into the corresponding Petri dish. Ten minutes later, once the specimen had acclimated to plate [4], we recorded the section (ceiling, wall, floor) where the fly tested was perched or moving.

In a second treatment, 4 nonvirgin females and 4 nonvirgin males *D*. *subobscura* remained within a Petri dish for 30 min. After this time, the adults were removed from the Petri dish, and one male (female) *D*. *melanogaster*, *D*. *simulans*, *D*. *immigrans* or *D*. *pavani* was deposited into the respective Petri dish with *D*. *subobscura* odors. Ten minutes later, we recorded the area (ceiling, wall, floor) where the fly tested was perched or moving. We used *D*. *subobscura* because after its arrival in Chile in 1980, the abundance of Chilean species of *Drosophila* decreased [33,34], suggesting that *D*. *subobscura* affected local *Drosophila* species. Preferences for sites to perch and moving of adult *D*. *subobscura* were tested in Petri dishes with their own odors and in Petri dishes with *D*. *pavani* odors.

In the two treatments described, after recording the section (ceiling, wall, floor) where each fly tested was perched or moving, we continued observing each imago for the next 2 min. We wanted to know whether the fly tested jumped from the area of the Petri dish (ceiling, wall, floor) where it had been found toward another section in the Petri dish.

The strains of *D*.*melanogaster* and *D*. *simulans* established with adults collected in apple orchards were not tested. By regulation, Jeannette Silva-López, undergraduate student at the Universidad del Bío-Bío, had a limited amount of time (10–12 months) to perform: i) open-field work, ii) experiments and iii) write a report on the results to obtain her Title of Teacher of Biology and Natural Sciences. These time constraints prevented testing all the strains.

### Patterns of movement

Adults aspirated into Petri dishes appeared more active in the presence of heterospecific than conspecific odors. We recorded movement patterns (locomotion and changes in direction) of adult *D*. *melanogaster*, *D*. *simulans*, *D*. *immigrans* and *D*. *pavani* in Petri dishes with conspecific odors and in Petri dishes with *D*. *subobscura* odors. Adults of this last species were tested against their own and *D*. *pavani* odors. We recorded the individual movement pattern for 2 minutes. We used a lucid Wild M5 camera. We made the records 10 min after an adult was introduced into the Petri dish. Some flies jumped from one sector (ceiling, wall, floor) to another before the end of the 2-min observation period. We discarded these flies and replaced them with new individuals of the same strain and species. In each drawing we recorded (i) the length of the trail using a Hoffritz Map measurer; we converted the distance obtained for each track into centimeters, and (ii) the size of the turning angles using a compass.

### Statistical analysis

#### Preferences for dark and illuminated sites

The data for preferences for dark, interface and light sites (Fig 4) were subjected to an analysis of variance. We tested adults of the Chillán and Quillón strains of *D*. *melanogaster*, *D*. *simulans*, *D*. *immigrans*, *D*. *subobscura*, and *D*. *pavani*. We applied a nested Model II ANOVA [35,36]. We also used the variances of preferences for light, dark, and interface zones and the sex x strain interaction to estimate the reliability of our records [36,37]. For this we calculate a correlation coefficient (details in [36,37]). A coefficient greater than 0.80 indicates that > 80% of the specimens tested chose between the dark, light, and interface areas of the boxes (Fig 4 and references [36,37]). We also estimated an index of concordance because J.S-L, an undergraduate student, did not have experience recording nominal behavioral events. Thus, we compared the number of agreements and disagreements between J. S-L and R. G-H regarding the assignment of the adults’ preferences for the light zone, the interface and the dark zone of the Petri dishes (Fig 4). We calculated a concordance index. This index was the proportion of all occurrences in which J.S-L and R.G-H coincided. We also organized the data obtained with each strain and species in a 2 x 3 (sex x sector of Petri dish) contingency table. We tested the hypothesis that the number of individuals in the dark zone, in the light zone and in the interface was independent of sex [36].

#### Perch and moving site selection

**Fig 5:**
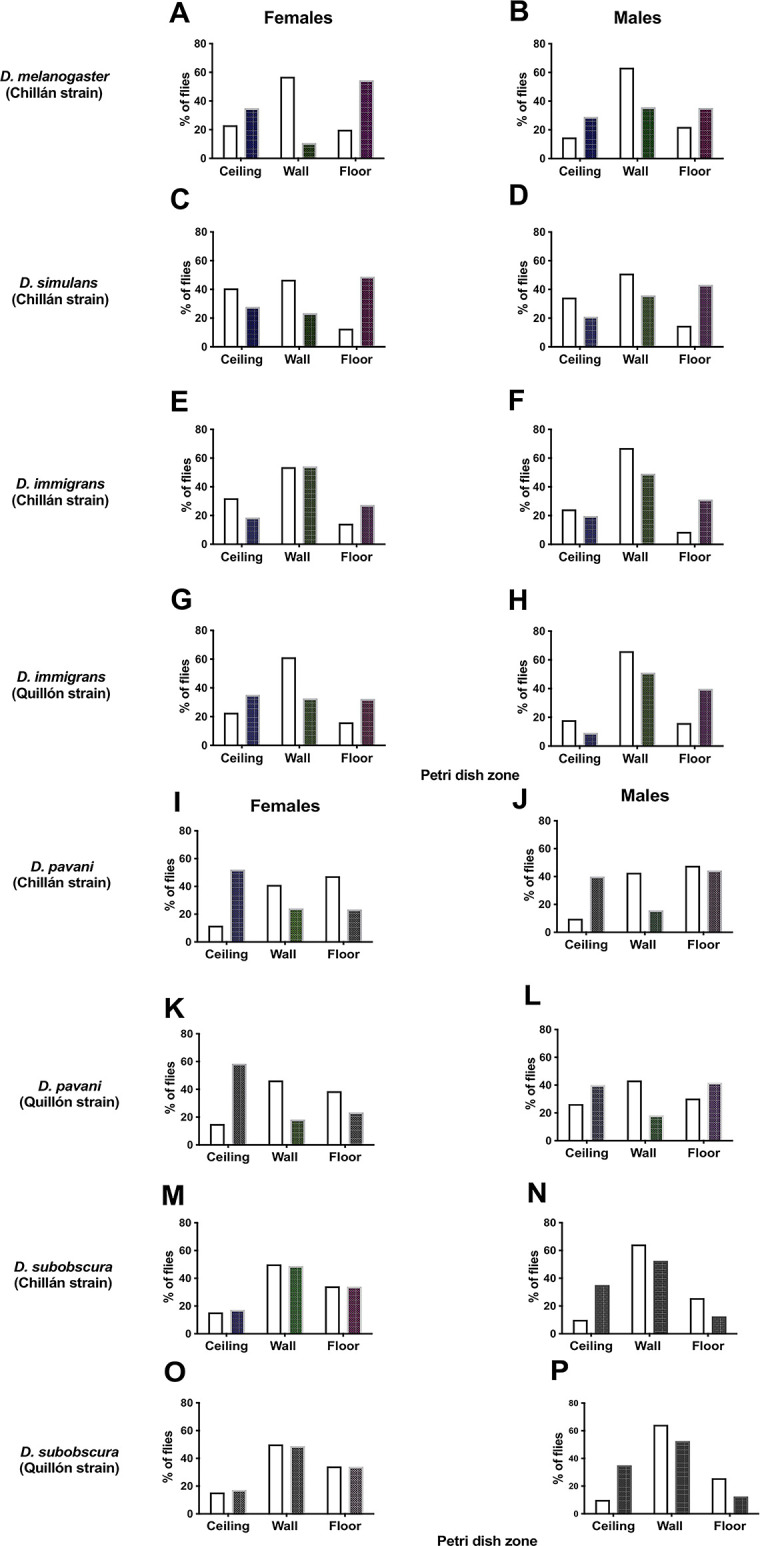
A–P. Preferences of adult *D*. *melanogaster* (A, B), *D*. *simulans* (C, D), *D immigrans* (E–H), *D pavani* (I–L), and *D*. *subobscura* (M–P) by site to perch/ move in Petri dishes with odors of the species (white columns) and in Petri dishes with odors of another *Drosophila* species (black columns). Preferences are expressed in percentages of flies (ordinate) on the ceiling, wall, and floor of the Petri dishes (abscissa). Adults *D*. *melanogaster*, *D*. *simulans*, *D*. *immigrans*, and *D*. *pavani* were tested against *D*. *subobscura* odors. This last species was tested against *D*. *pavani* odors.

For *D*. *melanogaster* and *D*. *simulans*, we tested only one strain (see Fig 5). Data obtained for each of these species were analyzed by applying a two-factor analysis of variance [36]. We evaluated the effects on perching and moving site preferences in Petri dishes with conspecific odors and *D*. *subobscura*. We also evaluated the sex of individuals tested, and the interaction between sex and odor treatment.

For data obtained with adult *D*. *immigrans*, *D*. *pavani* and *D*. *subobscura* we performed a multiway factorial analysis of variance because we recorded two strains per species. We evaluated the effects of i) conspecific and *D*. *subosbscura* odors (in the case of adult *D*. *subosbscura* we evaluated the effects of conspecific and *D*. *pavani* odors), ii) the sex of the individual tested, iii) the strain, iv) the interactions between two of these variables, and v) the sex x strain x odor treatment interaction.

#### Patterns of movement

A multiway ANOVA was applied to the data to test the significance of adult odors, species, strain and sex on i) length of the trail (estimator of locomotor activity) and ii) size of turn angles. The two behaviors are the principal components of adult *Drosophila* mobility [38,39]. We applied a correlation and regression analysis to compare the relationship between the length of the trail and the size of the angles made by the sexes in the presence of conspecific and heterospecific odors [36]. The length of the trail was chosen *a priori* as the independent variable because we thought that the adults showing a high rate of locomotion could also perform wider turning angles.

We used STATGRAPHICS Program, Centurion XVII 64-bit, 2014 version to make our statistical analysis.

## Results

### Fruit orchards

The apple and pear orchards consisted, each orchard, of 640 trees spread over 2 acres in each orchard (Fig 1). Between the trunks of adjacent apple (pear) trees there was a distance of 10.5 ± 0.50 m. At noon the canopy of each apple (pear) tree cast a shadow of 2.25 ± 0.33 m^2^ on the ground (n = 20 measurements/apple (pear) canopy/orchard/locality). The fruit orchards also had sunny areas (Fig 1). At noon the mean size of the sunny areas was 7.10 ± 0.18 m^2^ (n = 20 measurements/apple (pear) orchard/locality). The sun’s rays also passed through the canopies projecting onto the ground; the average size of these sunny/shady areas was 0.94 ± 0.12 m^2^ at noon (Fig 1). The size and shape of the shady, sunny, and sunny/shadow areas changed daily depending on the position of the sun in the sky.

At midday, the illumination in shady areas was approximately 420 ± 13 lux (n = 20 measurements/apple (pear) orchard/locality). In sunny places illumination was estimated to be approximately 50,000 ± 88 lux, whereas in the sunny/shady sites illumination was approximately 10,000 ± 19 lux (Fig 1).

### Temperatures in the orchards

The average environmental temperatures in sunny and shady habitats are in S1 Table (Chillán and Quillón localities). Measurements were made at midday. In shady places temperatures fluctuated around 24°C, whereas in sunny habitats reach over 40°C (S1 Table). Variances in shady sites are smaller than those calculated for sunny habitats (S1 Table).

### Fruits on the ground

In the pear and apple orchards the number of fruits on the ground per fruit tree fluctuated between 5 and 8. Thus, in each orchard the potential number of rearing sites for the five *Drosophila* species was 5 (number of fruit units) x 640 (number of fruit trees) = 3200 breeding sites. The average weight of a Fuji apple fallen on the ground was 175.00 ± 4.30 gr (n = 40 fruit units weighed). Therefore, under each apple tree there were an average of 175 x 5 = 875 gr of fruit (or approximately 0.88 kg of Fuji apple) available for the five *Drosophila* species. In the case of winter Nely pears, the mean weight per fruit unit was 216 ± 3.21 gr (n = 40 fruits weighed). That is under each pear tree there were on average 1.08 kg of pear available to the five *Drosophila* species.

### Distribution of adult *Drosophila* in the orchards

Between 9:00 am and 6:00 pm, adult *Drosophila* were in shady habitats. We did not find flies neither in sunny areas or in sunny/shady sections in the apple and pear orchards, Chillán and Quillón localities. In the shady areas, 75.42% of the adults of the five *Drosophila* species were on grasses and herbs at 8–10 cm from pear and apple fruits fallen on the ground. The sex ratio per species in grasses and herbs was close to 1.0 (S2 Table).

In apple and pear fruits fallen on the ground, also in shady places, we found mainly *D*. *simulans* and *D*. *subobscura* (S3 Table). Occasionally we detected *D*. *melanogaster*, *D*. *immigrans* and *D*. *pavani*. A total of 99.10% of adult *Drosophila* found in the fruits were females and 0.90% were males (S3 Table). The fruits showed few signs of fermentation (Fig 2), but they had fissures, presumably produced when they hit the ground. The adults were in the fissures (Fig 2).

### Ethological observations of adult *Drosophila* perched on grasses and herbs

On grasses and herbs in the shady areas of the fruit orchards there were groups of conspecifics of the five *Drosophila* species (Fig 3). Adults remained immobile and rarely left the cluster, suggesting that fly exchange between groups was sporadic or absent. The flies were attentive to environmental changes. They flew in all directions because of the disturbances caused by our arrival. We discovered the fly clusters because the dark-yellowish brown, pale yellow-black, and light reddish-black colors of the thorax and abdomen of the flies contrasted with the green color of the grasses and herbs on which they were perched. The groups were 8–10 cm from apple (pear) fruits fallen on the ground. We used a glass aspirator to carefully collect the groups of flies (*D*. *melanogaster*, *D*. *simulans*, *D*. *subobscura*, *D*. *immigrans* and *D*. *pavani*). Taxonomic classification confirmed that each cluster was composed of individuals of the same species (S2 Table). Although daily changes in the sun’s position modified the size and shape of shady sites, the fly groups were always detected in these habitats.

### Ethological observations on fruits fallen on the ground

Apples and pears lying on the ground had fissures with a depth of 1.5 to 3.0 mm and a length of 4.1 to 5.2 cm (Fig 2). The number of fissures per fruit (apple and pear) was 1.3 ± 0.2 on average. In the fissures mainly females of *D*. *simulans* and *D*. *subobscura* were found (S3 Table). The distance between females was approximately 1.0–1.9 cm (conspecific or heterospecific females). The females remained approximately 12.0 ± 1.4 min in the fissures. On the skin of the fruits, we found 1–2 adults on average distributed here and there (Fig 2). These individuals remained immobile on the pears for approximately 5.2 ± 0.7 min and then flew in different directions. Unlike adults perched on grasses and herbs, females in the fissures could be easily collected. The fruits did not show signs of bird pecking or rodent bites (Fig 2). We did not detect aggressive encounters between flies perched on fruits (peel and fissures), grasses and herbs.

### Experimental results

#### Response to illumination conditions

The variance ratios (*F*) resulting from individual preferences of males and females for the dark, interface and illuminated sections in the Petri dishes (Fig 4) were significant (*F*_*3*, *202*_ = 32.16, *P* < 0.05 for *D*. *pavani*; *F*_*3*, *202*_ = 32.56, *P* < 0.05 for *D*. *immigrans*; *F*_*3*, *202*_ = 38.14, *P* < 0.05 for *D*. *subobscura*; *F*_3, *202*_ = 41.56, *P* < 0.01 for *D*. *simulans*; and *F*_*3*,*202*_ = 42.02, *P* < 0.01 for *D*. *melanogaster*). The correlation coefficients calculated to estimate the reliability of the preferences of the sexes for illuminated, dark and interface areas (Table 1 and Fig 4) were as follow: 0.86 (*D*. *pavani*), 0.88 (*D*. *melanogaster*), 0.91 (*D*. *subobscura*), 0.93 (*D*. *simulans*), and 0.94 (*D*. *immigrans*). These values indicate that our method accounts for > 80% of adult *Drosophila* photo responses in the Petri dishes (Fig 4), and that < 20% represent errors of measurement, which is satisfactory for this kind of experiment [36,37]. The concordance index, the proportion of all occurrences about which J. S-L and R. G-H agreed, was 96.37%. Therefore, our method to measure the response of adult *Drosophila* to illumination conditions was reliable.

**Table 1:** Number of males and females *D*. *melanogaster*, *D*. *simulans*, *D*. *immigrans*, *D*. *subobscura* and *D*. *pavani* in the dark, illuminated and interface zones of Petri dishes (Fig 4). Two strains per species were tested. The strains were formed with individuals collected from shady habitats in pear orchards in Chillán and Quillón (details in Materials and Methods).

Species and strain	Petri dish zone							
	Dark		Illuminated		Interface		Total	
	♂	♀	♂	♀	♂	♀	♂	♀
*D*.*melanogaster*								
Chillán	30	42	5	–	15	8	50	50
Quillón	42	48	1	1	7	1	50	50
*D*. *simulans*								
Chillán	28	42	–	3	22	5	50	50
Quillón	28	39	12	1	10	10	50	50
*D*. *immigrans*								
Chillán	38	45	–	1	12	4	50	50
Quillón	43	47	–	2	7	1	50	50
*D*. *subobscura*								
Chillán	31	37	8	9	11	4	50	50
Quillón	34	40	1	6	15	4	50	50
*D*. *pavani*								
Chillán	50	48	–	1	–	1	50	50
Quillón	49	48	–	1	1	1	50	50
Total	373	436	27	25	100	39	500	500

Table 1 shows that 373 out of 500 (74.60%) males and 436 out of 500 (87.20%) females of the Chillán and Quillón strains of *D*. *melanogaster*, *D*. *simulans*, *D*. *immigrans*, *D*. *subobscura*, and *D*. *pavani* were in the dark sector of Petri dishes, whereas 27 out of 500 (5.40%) males and 25 out of 500 (5.00%) females of the same strains and species were in the illuminated zone of Petri dishes. On the other hand, 100 out of 500 (20.00%) males and 39 out of 500 (7.80%) females of the species and strains were in the interface zone (Table 1). The chi-square goodness of fit values for the number of males in the dark, illuminated and interface zones for species and strains indicated in Table 1 were all greater than the critical value χ^**2**^_**0.05(2)**_ = 5.99. Similar results were obtained with the females (Table 1) and the chi-square goodness of fit values were also greater than the critical value χ^**2**^_**0.05(2)**_ = 5.99.

Taken as a whole these results point to a general preference for shade places.

We also tested the hypothesis that the number of individuals in the dark, illuminated and interface areas of the Petri dishes was independent of sex. In the two strains of *D*. *simulans*, numbers of females and males in the dark, illuminated and interface zones of the Petri dishes were significantly different: χ^2^ = 11.48, the Chillán strain and χ^2^ = 21.61, the Quillón strain, *df* = 2, *P* < 0.005. An examination of the data showed that the number of females in the dark zone exceeded the expected value (Table 1).

In *D*. *melanogaster*, *D*. *subobscura* and *D*. *immigrans*, only one population exceeded the expected frequency of females in the dark zone of the Petri dishes (Table 1): χ^2^ = 11.06, *df* = 2, *P* < 0.01, *D*. *melanogaster*, the Chillán strain; χ^2^ = 9.27, *df* = 2, *P* < 0.01, *D*. *immigrans*, the Chillán strain; and χ^2^ = 11.96, *D*. *subobscura*, the Quillón strain, *df* = 2, *P* < 0.01. The sexes of the Chillán and Quillón strains of endemic *D*. *pavani* showed similar preferences for the dark zone of the Petri dishes; χ^2^ = 1.56, the Chillán strain; and χ^2^ = 0.53, the Quillón strain, *df* = 2, *P* > 0.05.

**Perch site selection.** Fig 5A–P shows percentages of adults *D*. *melanogaster* (A, B), *D*. *simulans* (C, D), *D immigrans* (E–H), *D pavani* (I—L) and *D*. *subobscura* (M–P) perched/moving in sectors of Petri dishes (celling, wall and floor). Adults *D*. *melanogaster*, *D*. *simulans*, *D immigrans*, and *D pavani* were tested in Petri dishes with odors of the species (white columns) and in Petri dishes with odors of *D*. *subobscura* (black columns). This last species was tested against conspecific odors and *D*. *pavani* odors. Data show the Chillán strains of *D*. *melanogaster* and *D*. *simulans* formed with adults collected in the pear orchard. For the other *Drosophila* species, the strains tested were formed with individuals collected in the pear orchards in Chillán and Quillón.

Fig 5A and 5B shows that in the Petri dishes with conspecific odors the sexes of *D*. *melanogaster* prefer the wall to perch/move (white columns). In petri dishes with odors of *D*. *subobscura*, female *D*. *melanogaster* prefer perch/move on the floor and ceiling (Fig 5A, black columns), whereas the males perch/move on the wall, floor, and ceiling (Fig 5B, black columns). The differences between the sexes in response to *D*. *subobscura* odors were significant (*F*_***0*.*05(1*,*93)***_ = 4.57 for the interaction of sex and odor treatment; *P* < 0.05).

Fig 5C and 5D shows that the sexes *D*. *simulans* prefer to perch/move on the ceiling and wall in Petri dishes with conspecific odors (white columns). In Petri dishes with *D*. *subobscura* odors (black columns), the sexes *D*. *simulans* prefer perch/move on the floor (*F*_***0*.*05(1*,*93)***_ = 4.33 for odor treatment; *P* < 0.05). The interaction of sex and odor treatment for the selection of site to perch/move was not statistically significant (*F*_***0*.*05(1*,*93)***_ = 2. 31; P > 0.05).

Fig 5E–5H shows that approximately 60% of adults of the Chillán and Quillón strains of *D*. *immigrans* choose perch/move on the wall of Petri dishes with conspecific odors (white columns). In Petri dishes with *D*. *subobscura* odors, the sexes of the Chillán strain prefer perch/move on the wall and floor (Fig 5E and 5F; black columns). In contrast, Quillón females of *D*. *immigrans* choose perch/move on the ceiling, wall, and floor in Petri dishes with *D*. *subobscura* odors (Fig 5G; black columns), whereas the males choose perch/move on the wall and floor (Fig 5H; black columns). The sex x strain x odor treatment interaction on perch/move site preferences of adult *D*. *immigrans* was statistically significant (*F*_**0.05(1,192)**_ = 4.19; *P* < 0.05).

Adults of the Chillán and Quillón strains of *D*. *pavani* choose the wall and floor to perch/move in Petri dishes with conspecific odors (Fig 5I–5L; white columns). In the presence of *D*. *subobscura* odors (black columns), females of the two *D*. *pavani* strains prefer perch/move on the ceiling (Fig 5I and 5K), whereas the males perch/move on the ceiling and floor (Fig 5J and 5L). The interaction sex x strain x odor treatment on the perch/move sites preferences of adult *D*. *pavani* was statistically important (*F*_**0.05(1,192)**_ = 4.62; *P* < 0.05).

In Petri dishes with conspecific odors, adults of the Chillán and Quillón strains of *D*. *subobscura* choose perch/move on the wall (Fig 5M–5P; white columns). Stimulated by *D*. *pavani* odors, females of the two strains choose the wall and floor to stay/move (Fig 5M and 5O), and the males prefer the ceiling and wall (Fig 5N and 5P). The interaction sex x strain x odor treatment on the perch site preferences of adult *D*. *subobscura* was statistically significant (***F***_**0.05(1,192)**_ = 4.07; *P* < 0.05).

After recording the sector (ceiling, wall, floor) in a Petri dish on which a fly was, we observed the specimen for another 2 min. We recorded if the fly remained in the same sector or jumped to another place in the Petri dish. In the Petri dishes with conspecific odors, not more than 6.0% of the sexes jumped toward other sections (Table 2). In Petri dishes with odors of another *Drosophila* species at least 12.0% of the flies jumped to other sections (Table 2). These differences are statistically important (the *G* test for independence showed that the calculated *Gs* were all higher than the critical value χ^2^_0.05(1)_ = 3.84).

**Table 2:** Number and percentage of males and females *D*. *melanogaster*, *D*. *simulans*, *D*. *immigrans*, *D*. *pavani* and *D*. *subobscura* jumping from one sector toward another one in Petri dishes. The capsules had conspecific odors or odors of another *Drosophila* species (details in Materials and Methods).

	Petri dishes with							
Species and strain	odors of conspecifics				odors of another *Drosophila* species^a^			
	♂		♀		♂		♀	
	N°	%	N°	%	N°	%	N°	%
*D*.*melanogaster*								
Chillán	2	4.00	1	2.00	15	30.00	16	32.00
*D*. *simulans*								
Chillán	–	–	–	–	11	22.00	10	20.00
*D*. *immigrans*								
Chillán	–	–	–	–	10	20.00	13	26.00
Quillón	1	2.00	1	2.00	12	24.00	12	24.00
*D*. *pavani*								
Chillán	1	2.00	1	2.00	9	18.00	9	18.00
Quillón	3	6.00	–	–	10	20.00	10	20.00
*D*. *subobscura*								
Chillán	–	–	–	–	6	12.00	8	16.00
Quillón	–	–	–	–	9	18.00	9	18.00
Total flies jumping	7	–	3	–	82	–	87	–

^**a**^For adult *D*. *melanogaster*, *D*. *simulans*, *D*. *immigrans* and *D*. *pavani* heterospecific odors were *D*. *subobscura*. For this last species heterospecific odors were *D*. *pavani*.

#### Patterns of movement

The mobility of adult *Drosophila* entails mainly locomotion and turns [38,39]. Table 3 shows the two activities for males and females of the Chillán and Quillón strains of *D*. *melanogaster*, *D*. *simulans*, *D*. *immigrans*, *D*. *pavani* and *D*. *subobscura* in Petri dishes with conspecific and heterospecific odors. Adults of the Chillán and Quillón strains of *D*. *melanogaster*, *D*. *simulans*, *D*. *immigrans*, and *D*. *pavani* increase locomotion and the size of turning angles in Petri dishes with *D*. *subobscura* odors and decrease these behaviors in Petri dishes with conspecific odors (Table 3). Adults of the Chillán and Quillón strains of *D*. *subobscura* also decreased locomotion and size of turns in Petri dishes with conspecific odors and increased the behaviors in Petri dishes with *D*. *pavani* odors (Table 3). The interaction of sex x strain x species x odor treatment on locomotion and turning amplitude was statistically significant (***F***_**0.05(4, 35)**_ = 7.42; *P* < 0. 0005).

**Table 3:** Locomotion and turning angle sizes of adult *D*. *melanogaster*, *D*. *simulans*, *D immigrans*, *D*. *subobscura* and *D pavani* in Petri dishes with conspecific and strange odors^a^. n = 50 individuals per sex/strain/species were scored. The strains tested were formed with adults collected in pear orchards in Chillán and Quillón localities (see Materials and Methods).

Species, strain and sex	conspecific odors		strange odors^a^	
	locomotion (cm/2 min) Mean ± SE	turning angle sizes (grades) Mean ± SE	locomotion (cm/2 min) Mean ± SE	turning angle size (grades) Mean ± SE
1) *D*. *melanogaster*				
Chillán				
Male	3.03 ± 0.39	34.63 ± 4.71	5.48 ± 0.28	55.49 ± 4.01
Female	4.33 ± 0.30	50.05 ± 4.00	5.91 ± 0.21	58.37 ± 3.30
Quillón				
Male	1.85 ± 0.17	21.21 ± 3.88	3.84 ± 0.37	41.25 ± 1.17
Female	3.38 ± 0.36	45.93 ± 5.12	4.73 ± 0.30	47.14 ± 3.70
2) *D*. *simulans*				
Chillán				
Male	0.46 ± 0.10	4.96 ± 1.99	4.17 ± 0.32	31.78 ± 3.98
Female	0.24 ± 0.06	0.23 ± 0.23	4.80 ± 0.36	37.48 ± 4.07
Quillón				
male	4.03 ± 0.31	24.06 ± 1.99	5.92 ± 0.32	58.01 ± 2.39
female	4.07 ± 0.28	52.25 ± 3.83	6.98 ± 0.90	62.47 ± 3.42
3) *D*.*immigrans*				
Chillán				
male	3.82 ± 0.37	41.05 ± 3.93	4.90 ± 0.32	48.60 ± 4.00
female	4.50 ± 0.34	46.40 ± 4.21	5.23 ± 0.32	50.50 ± 4.40
Quillón				
male	1.10 ± 0.25	10.42 ± 3.13	1.46 ± 0.27	14.72 ± 3.47
female	2.44 ± 0.33	26.70 ± 4.58	3.77 ± 0.34	41.42 ± 5.13
4) *D*. *subobscura*				
Chillán				
male	0.09 ± 0.04	0.00 ± 0.00	3.80 ± 0.27	96.80 ± 3.65
female	0.74 ± 0.21	5.80 ± 2.44	3.37 ± 0.36	23.98 ± 3.46
Quillón				
male	3.88 ± 0.28	46.12 ± 4.29	5.46 ± 0.17	54.46 ± 3.13
female	4.35 ± 0.27	44.83 ± 3.80	5.61 ± 0.20	57.87 ± 2.90
5) *D*. *pavani*				
Chillán				
male	2.67 ± 0.38	23.72 ± 2.09	3.89 ± 1.54	31.22 ± 3.81
female	4.42 + 2.31	17.50 + 1.32	7.43 + 1.32	38.65 + 2.92
Quillón				
male	1.03 ± 0.22	29.71 ± 2.76	5.86 ± 0.67	40.11 ± 2.03
female	3.60 ± 1.03	12.74 + 0.34	8.06 ± 1.64	54.71 ± 3.15

^**a**^For adult *D*. *melanogaster*, *D*. *simulans*, *D*. *immigrans* and *D*. *pavani* heterospecific odors corresponded to odors emitted by adult *D*. *subobscura*. For this last species heterospecific odors were emitted by adult *D*. *pavani*.

Table 4 shows Pearson correlation coefficients and slopes of linear regression for angle size on adult locomotion in the Chillán and Quillón strains of *D*. *melanogaster*, *D*. *simulans*, *D*. *immigrans* and *D*. *pavani* in the presence of conspecific and *D*. *subobscura* odors. Adults of this latter species were tested in the presence of conspecific and *D*. *pavani* odors. There were statistically significant positive correlations among the two behaviors of males and females of the strains and species (***F***_**0.05 (45,50)**_ values were: 2.44, *P* < 0.005, *D*. *melanogaster*; 2.93, *P* < 0.005, *D*. *simulans*; 2.67, *P* < 0.05, *D*. *immigrans*; 2.71, *P* < 0.005, *D*. *subobscura*; and 2.41, *P* < 0.005, *D*. *pavani*; ***F***_**0.05 (45,50**)_ critical value = 1.68, *P* = 0.05). There is also significant linearity between size of turns and locomotion (|*t*_*(98)*_| values were: 4.56, *D*.*melanogaster*; 5.01, *D*. *simulans*; 4.28, *D*. *immigrans*; 4.78, *D*. *subobscura*, and 4.22, *D*. *pavani*; *t*_(98)_ = 3.18 critical value, *P* < 0.001). The findings suggest that conspecific odors and odors emitted by other *Drosophila* modulate mobility in the strains and species tested.

**Table 4:** Correlation and regression analysis of angle size on the locomotion from adult *D*. *melanogaster*, *D*. *simulans*, *D immigrans*, *D*. *subobscura* and *D pavani* confronted with conspecific and strange odors. The strains were collected in Chillán and Quillón. See also Table 3 and Materials and Methods.

Species, strain and sex	Pearson correlation coefficient (a ± SE)		Slope of linear regression (b ± SE)	
	Conspecific odors	Strange odors	Conspecific odors	Strange odors
1) *D*. *melanogaster*				
Chillán				
male	0.81 ± 0.02	0.75 ± 0.06	0.69 ± 0.01	0.76 ± 0.07
female	0.84 ± 0.04	0.64 ± 0.08	0.65 ± 0.12	0.81 ± 0.02
Quillón				
male	0.74 ± 0.02	0.71 ± 0.01	0.68 ± 0.16	0.88 ± 0.04
female	0.64 ± 0.06	0.78 ± 0.03	0.75 ± 0.10	0.91 ± 0.02
2) *D*. *simulans*				
Chillán				
male	0.67 ± 0.01	0.72 ± 0.06	0.62 ± 0.16	0.96 ± 0.03
female	0.69 ± 0.01	0.73 ± 0.08	0.63 ± 0.19	0.87 ± 0.04
Quillón				
male	0.58 ± 0.01	0.65 ± 0.07	0.62 ± 0.11	0.88 ± 0.09
female	0.72 ± 0.05	0.71 ± 0.01	0.72 ± 0.09	0.89 ± 0.03
3) *D*.*immigrans*				
Chillán				
male	0.69 ± 0.01	0.73 ± 0.08	0.73 ± 0.19	0.87 ± 0.04
female	0.66 ± 0.03	0.63 ± 0.11	0.77 ± 0.09	0.81 ± 0.08
Quillón				
male	0.61 ± 0.06	0.76 ± 0.12	0.68 ± 0.03	0.78 ± 0.11
female	0.76 ± 0.13	0.67 ± 0.01	0.53 ± 0.05	0.71 ± 0.10
4) *D*. *subobscura*				
Chillán				
male	0.59 ± 0.02	0.58 ± 0.01	0.67 ± 0.09	0.71 ± 0.08
female	0. 53 ± 0.03	0.61 ± 0.06	0.61 ± 0.02	0.81 ± 0.10
Quillón				
male	0. 59 ± 0.05	0.60 ± 0.04	0.65 ± 0.01	0.73 ± 0.05
female	0. 60 ± 0.04	0.55 ± 0.03	0.66 ± 0.04	0.83 ± 0.05
5) *D*. *pavani*				
Chillán				
male	0. 56 ± 0.01	0.65 ± 0.03	0.58 ± 0.02	0.71 ± 0.08
female	0. 58 ± 0.06	0.57 ± 0.02	0.60 ± 0.07	0.77 ± 0.05
Quillón				
male	0. 61 ± 0.05	0.62 ± 0.04	0.68 ± 0.07	0.84 ± 0.06
female	0. 57 ± 0.01	0.61 ± 0.05	0.63 ± 0.07	0.75 ± 0.05

For the significance of the Pearson correlation coefficients, the calculated *F*_**0.05(50,45)**_ values were all greater than the critical value *F*_**0.05(50,45)**_ = 2.44. The significance of differences between slopes of linear regression within strains in the presence of conspecific odors versus strange odors yielded *t*_*(98)*_ values greater than *t*_*(98)*_ > 3.175 critical value, *P* < 0.001. The analysis indicates that the relationship between locomotion and angle size is maintained in the presence of conspecific and heterospecific odors, but slopes of the linear regression are greater in the presence of heterospecific odors. Our findings are in general agreements with those reported by York et.al. [39].

## Discussion

We focused on aspects of nonsexual behavior of adults of five *Drosophila* species in pear and apple orchards. These fruit orchards had contrasting habitats that differed in illumination conditions: i) sunny, ii) shady, and iii) sunny/shady sites (Fig 1). Adults of *D*. *melanogaster*, *D*. *simulans*, *D*. *immigrans*, *D*. *subobscura*, and the endemic *D*. *pavani* occupied shady sections. These species formed conspecific aggregations on the ground (Fig 3). On the fruits there were no conspecific groups and most of the adults were females (S3 Table). They were in the fissures exhibited by the fruits (Fig 2); in the same fissure there could be females of two *Drosophila* species (S3 Table). Thus, the social interactions between the sexes of a species and between *Drosophila* species depend on the microenvironment they occupy. Finding mainly females in the fruits suggests an evolutionary change between the sexes of the five *Drosophila* species relative to the occupation of food sources.

Our experimental results agree with our observations on the adult *Drosophila* behavior in the fruit orchards. That is, the adults preferred dark sites to rest and move (Fig 4 and Table 1). We conjecture that i) changing lighting conditions in orchards modifying the size and shape of shady sites and ii) adult preferences for dimly lit sites influence daily movements and relocations of imagoes of the five species toward new shady sections. Higher temperatures in sunny habitats could also encourage adults to move toward shady areas [10]. Dimly lit sectors have more stable temperatures (S1 Table) and presumably higher humidity than sunny areas. Temperature and humidity influence *Drosophila* physiology [40]. Therefore, the shady sites could provide stability to the development of the five *Drosophila* species.

Our data also suggest that odors emanating from adults of the five species matter in space occupation in the fruit orchards. Thus, odors of one species i) decrease mobility and ii) favor the conspecifics selecting similar sites to perch/move (Tables 2–4, and Fig 5A–5P, white columns). Those same odors in another species i) increase adult mobility (Tables 2–4) and ii) influence each sex to choose a different perching site (Fig 5A–5P, black columns). Odors emitted by the adults of a *Drosophila* species decreasing mobility in their congeners and stimulating similar preferences for perching sites could be the first steps in group formation. That these same odors increase mobility and change perch site preferences in each one of the sexes of other *Drosophila* species could hinder the formation of mixed groups composed of two or more species. These conjectures are in alignment with it being difficult to find interspecific hybrids of *Drosophila* in nature [41]. However, *Drosophila yakuba* females and *Drosophila teissieri* males originate F1 progeny in nature. These hybrids exhibit, however, great reproductive and physiological deficiencies incompatible with life [42].

Why were most of the adults of the five *Drosophila* species in grasses and herbs and absent in fruits? Why were most of the individuals found in the fruit females? (S3 Table). Assuming that the females detected in apples and pears were nonvirgin, they could be laying their eggs. We conjectured that the odors given off by decaying fruits could attract nonvirgin females repelling males and virgin females. Fruit odors modify *Drosophila* social behavior [43,44]. Laying eggs in the fissures of apples and pears might help the larvae hatched from the eggs gain access to food. The absence of males and virgin males and females could be logical; they could interfere with egg laying activity. Future work should test these conjectures.

The five *Drosophila* species belong to different clades [23]. However, adults exhibit a similar ability and willingness to integrate responses to illumination conditions with reactions to odors from conspecifics and strangers. These findings suggest the conservation of genetic activity and neurological coordination for the integration of routines in all five species.

## Supporting information

S1 TableTemperatures (°C), $\bar{x}$ ± SE, recorded in sunny and shady sites at the apple and pear orchards where adult *D*. *melanogaster*, *D*. *simulans*, *D*. *immigrans*, *D*. *subobscura* and *D*. *pavani* were collected.Mean temperatures values and their corresponding variances were recorded in the time range 12.30–2.30 pm. Measurements were made in N = 40 sunny (shady) places in each orchard (details in Materials and Methods).(PDF)

S2 Table Abundance of males and females *D*. *melanogaster*, *D*. *simulans*, *D*. *immigrans*, *D*. *subobscura* and *D*. *pavani* in apple and pear orchards located in Chillán and Quillón. The orchards had sunny, shady and sunny/shady places. Individuals listed below were perched on grasses and herbs in shady habitats in the Chillán and Quillón orchards. In sunny and sunny/shady areas adult *Drosophila* was not detected. The total number of individuals per species/orchard/locality is shown. In parentheses is the number of groups of flies per species/fruit orchard/locality (see Results).
(PDF)

S3 Table Number of females and males *D*. *simulans* and *D*. *subobscura* found in fissures exhibited by apple and pear fruits fallen on the ground. The fruits were in shady environments (see Materials and Methods). The adults were collected between 9.0 am and 6.0 pm.
(PDF)
