# Supplementary material for: Interaction and integration among behaviors of adult Drosophila in nature
Source: PLoS One. 2023 Jul 13;18(7):e0278427. doi: 10.1371/journal.pone.0278427 (PMC10343093; doi:10.1371/journal.pone.0278427)
Supplement: S1 Table — Mean temperatures values and their corresponding variances were recorded in the time range 12.30–2.30 pm. Measurements were made in N = 40 sunny (shady) places in each orchard (details in Materials and Methods). (PDF) [file pone.0278427.s001.pdf]

S1 Table. Temperatures (ºC), $\bar{x} \pm$ SE, recorded in sunny and shady sites at the apple and pear orchards where adult *D. melanogaster*, *D. simulans*, *D. immigrans*, *D. subobscura* and *D. pavani* were collected. Mean temperatures values and their corresponding variances were recorded in the time range 12.30 – 2.30 pm. Measurements were made in N = 40 sunny (shady) places in each orchard (details in Materials and Methods).

	Temperature (ºC)							
	Apple orchard				Pear orchard			
	Sunny places		Shady places		Sunny places		Shady places	
Locality	$\bar{x} \pm$ SE	S^2	$\bar{x} \pm$ SE	S^2	$\bar{x} \pm$ SE	S^2	$\bar{x} \pm$ SE	S^2
Chillán	38.43±1.59	26.35	24.05 ±0.12	0.67	44.78±2.19	39.68.	25.78 ±0.14	1.00
Quillón	41.51±1.64	34.38	24.19±0.01	0.10	47.51±1.39	56.60	24.30±2.00	4.04

Mean temperatures in sunny places located in the Quillón orchards tend to be greater than those in shady places in the Chillán and Quillón orchards. Shady places in the Chillán and Quillón orchards have similar mean temperatures. Variances for temperature computed for sunny areas are greater than the calculated for shady habitats.
