# Supplementary material for: Interaction and integration among behaviors of adult Drosophila in nature
Source: PLoS One. 2023 Jul 13;18(7):e0278427. doi: 10.1371/journal.pone.0278427 (PMC10343093; doi:10.1371/journal.pone.0278427)
Supplement: S2 Table — The orchards had sunny, shady and sunny/shady places. Individuals listed below were perched on grasses and herbs in shady habitats in the Chillán and Quillón orchards. In sunny and sunny/shady areas adult Drosophila was not detected. The total number of individuals per species/orchard/locality is shown. In parentheses is the number of groups of flies per species/fruit orchard/locality (see Results). (PDF) [file pone.0278427.s002.pdf]

S2 Table. Abundance of males and females *D. melanogaster*, *D. simulans*, *D. immigrans*, *D. subobscura* and *D. pavani* in apple and pear orchards located in Chillán and Quillón. The orchards had sunny, shady and sunny/shady places. Individuals listed below were perched on grasses and herbs in shady habitats in the Chillán and Quillón orchards. In sunny and sunny/shady areas adult *Drosophila* was not detected. The total number of individuals per species/orchard/locality is shown. In parentheses is the number of groups of flies per species/fruit orchard/locality (see Results).

Species, and locality	Orchards						Total flies per site
	Apple		Pear		Total per sex		
	♂	♀	♂	♀	♂	♀	
D. melanogaster							
Chillán	15	19 (3)	6	9 (1)	21	28	49
Quillón	14	12 (2)	5	7 (1)	19	19	36
D. simulans							
Chillán	50	58 (5)	50	55 (5)	100	113	213
Quillón	76	72 (9)	63	67 (6)	139	139	278
D. immigrans							
Chillán	3	5 (1)	2	1 (1)	5	6	11
Quillón	8	7 (1)	3	2 (1)	11	9	20
D. subobscura							
Chillán	41	49 (4)	38	45 (5)	79	94	173
Quillón	63	69 (4)	43	47 (3)	106	116	222
D. pavani							
Chillán	2	1 (1)	-	1	2	2	4
Quillón	2	3 (1)	2	7 (1)	4	10	14
Grand Total	274	295	212	241	486	536	1022

In the apple and pear orchards located in Chillán and Quillón about 86.69 % of the flies belonged to *D. simulans* and *D. subobscura*, and 13.31% to *D. melanogaster*, *D. immigrans* and *D. pavani*. The least abundant is Chilean endemic *D. pavani* (1.76 %).
