# Supplementary material for: Interaction and integration among behaviors of adult Drosophila in nature
Source: PLoS One. 2023 Jul 13;18(7):e0278427. doi: 10.1371/journal.pone.0278427 (PMC10343093; doi:10.1371/journal.pone.0278427)
Supplement: S3 Table — The fruits were in shady environments (see Materials and Methods). The adults were collected between 9.0 am and 6.0 pm. (PDF) [file pone.0278427.s003.pdf]

S3 Table. **Number of females and males *D. simulans* and *D. subobscura* found in fissures exhibited by apple and pear fruits fallen on the ground.** The fruits were in shady environments (see Materials and Methods). The adults were collected between 9.0 am and 6.0 pm.

Locality, species and fruit	Nº of adults in fruit fissures		Total flies	Nº fruits examined
	♂	♀		
Chillán				
D.simulans				
Apple	1	50	51	20
Pear	-	56	56	22
D. subobscura				
Apple	-	21	21	20
Pear	2	22	24	22
Quillón				
D. simulans				
Apple	-	61	61	28
Pear	-	78	78	24
D. subobscura				
Apple	-	27	27	28
Pear		15	15	24
Grand Total	3	330	333	94

Adult *D. melanogaster*, *D. immigrans* and *D. pavani* were not observed in the fruit samples. Number of fruits observed was 94. On the fruit fissures (apples and pears) were detected in average 2.61 $\pm$ 1.02 females and 0.01$\pm$ 0.01 males (*D. simulans*), and 0.90 $\pm$ 0.02 females and 0.02 $\pm$ 0.01 males (*D. subobscura*). See also Fig 2.
